# A Visit to Bethlem Hospital

**Published:** 1919-12-06

**Authors:** 


### A Visit to Bethlem Hospital.

HOGARTH, BUNYAN, AND TWO BOMBS.

THE other day an interested party connected with the London and Country Rambling Society, which is keen on historical research, gathered by invitation at the hospital and heard a lecture by the Chaplain. It was news to most of us that the magnificent building in Lambeth was twice bombed during the war. The slides thrown on the screen showed that one bomb made a big crater in the garden, and another smashed many windows. Happily there were no casualties. The Chaplain had a good deal to say about the unhappy scenes of bygone days, when Hogarth depicted the horrors of "the madhouse" at Moorfields, and when the patients provided amusement for holiday promenaders. The better days were brought about largely by the Quaker, William Tuke, who began the humane treatment of the present day. Now the patients, who have entertainments in their theatre twice a week, are greatly enjoying dancing. An out-patient department was recently opened—houses in which every suitable case is treated in such a way as to prevent the patient having to enter the institution.

The Chaplain not only described the 672 years of the hospital's history, on which he has written a remarkable book, but aroused the interest of the visitors by expressing the opinion that the *Pilgrim's Progress* was an account of Bunyan's insanity—a theory which is a good deal supported by the fact that Bunyan is thought to have suffered from religious melancholia.

